# 2-*C*-Hydro­xymethyl-2,3-*O*-iso­propyl­idene-d-ribono-1,5-lactam. Corrigendum

**DOI:** 10.1107/S1600536810043096

**Published:** 2010-11-23

**Authors:** Christopher R. Newton, Michela Iezzi Simone, George W. J. Fleet, Yves Blériot, David J. Watkin

**Affiliations:** a Department of Chemical Crystallography, Chemistry Research Laboratory, Oxford OX1 3TA, England; b Department of Organic Chemistry, Chemistry Research Laboratory, Oxford OX1 3TA, England; c Ecole Normale Supérieure, Département de Chimie, UMR 8642, 24 rue Lhomond, 75231 Paris Cedex 05, France

## Abstract

The name of one of the authors in the paper by Newton *et al.* [*Acta Cryst.* (2004), E**60**, o909–o910] is corrected.

## Abstract 2

Corrigendum to *Acta Cryst.* (2004), E**60**, o909–o910.

In the paper by Newton *et al.* (2004) ▶, the second author is incorrectly given as ‘Iezzi Simone Michela’. The correct name should be ‘Michela Iezzi Simone’, as given above.
